# Methyltransferase‐Like 14 Promotes the Angiogenesis of Hepatocellular Carcinoma via Targeting Vascular Endothelial Growth Factor A

**DOI:** 10.1002/cnr2.70592

**Published:** 2026-05-29

**Authors:** Xinmiao Xiong, Chuanfang Shu, Ziqun Qu, Jiayan Li, Wei Ye, Lili Wang

**Affiliations:** ^1^ Department of Infectious Disease The Second Hospital of Nanjing, Nanjing University of Chinese Medicine Nanjing Jiangsu China; ^2^ The Clinical Infectious Disease Center of Nanjing Nanjing Jiangsu China; ^3^ Clinical Research Center The Second Hospital of Nanjing, Nanjing University of Chinese Medicine Nanjing Jiangsu China

**Keywords:** angiogenesis, hepatocellular carcinoma, METTL14, post‐transcriptional modification, VEGFA

## Abstract

**Background:**

N^6^‐methyladenosine (m^6^A) modification is a pivotal epitranscriptomic regulator implicated in tumor progression. As a core component of the m^6^A methyltransferase complex, METTL14 is known to suppress tumorigenesis; however, its specific role in tumor angiogenesis remains elusive. Given that Vascular Endothelial Growth Factor A (VEGFA) serves as the dominant driver of angiogenesis, we hypothesized that METTL14 may govern VEGFA expression to modulate the vascular niche in hepatocellular carcinoma (HCC).

**Aims:**

This study aimed to elucidate the regulatory axis between METTL14 and VEGFA in HCC. Specifically, we sought to determine whether METTL14 functions as a suppressor of HCC progression by restricting VEGFA‐driven angiogenesis through an m^6^A‐dependent mechanism.

**Methods:**

Quantitative m^6^A levels were performed to compare modification levels between HCC tissues and paired normal tissues. Bioinformatics analysis was performed to investigate the association between METTL14 and VEGFA expression levels and overall survival (OS) in a cohort of 364 patients with HCC. Immunohistochemistry was conducted to evaluate METTL14 expression in clinical specimens. Functional assays (CCK‐8, colony formation, and Transwell) were utilized to assess the effects of METTL14 modulation on cell proliferation and metastasis. Western blot analysis confirmed the regulatory effect of METTL14 on VEGFA protein levels. In vitro tube formation assays and in vivo Matrigel plug assays were performed to evaluate angiogenic capacity. Mechanistic insights were gained using MeRIP‐qPCR and RNA Pull‐Down Assays to identify m^6^A modification sites on VEGFA mRNA.

**Results:**

m^6^A levels and METTL14 expression were significantly downregulated in HCC tissues. METTL14 knockdown promoted HCC cell proliferation and invasion, whereas overexpression suppressed these phenotypes. Functionally, METTL14 depletion promoted HUVEC tube formation in vitro and augmented tumor angiogenesis in vivo, effects mechanistically linked to VEGFA upregulation. Mechanistically, METTL14 directly bound to and deposited m^6^A modifications on VEGFA mRNA, leading to its destabilization and subsequent degradation. Consequently, reduced METTL14 expression resulted in the sustained upregulation of VEGFA in HCC.

**Conclusion:**

METTL14 functions as a critical tumor suppressor in HCC by restricting VEGFA‐driven angiogenesis via m^6^A‐dependent mRNA destabilization. Our findings uncover a novel epigenetic regulatory mechanism in HCC and identify METTL14 as a potential therapeutic target for anti‐angiogenic intervention.

## 1 Introduction

Hepatocellular carcinoma (HCC) represents a formidable global health burden, ranking as the sixth most common malignancy worldwide (accounting for 4.7% of all cancer cases) and the third leading cause of cancer‐related mortality (8.3% of cancer deaths) [1]. Angiogenesis is fundamental to tumor growth, invasion, and metastasis [2, 3]. Currently, antiangiogenic monotherapy or combination regimens with immune checkpoint inhibitors have emerged as a cornerstone of antitumor therapy for HCC [2, 4, 5, 6]. However, only a subset of patients responds to antiangiogenic monotherapies (e.g., sorafenib, regorafenib, lenvatinib, bevacizumab) [7], and frequent development of drug resistance following initial response limits their long‐term efficacy [8]. These observations highlight that the molecular underpinnings of tumor angiogenesis remain incompletely elucidated, underscoring the need for deeper mechanistic exploration.

N6‐methyladenosine (m^6^A) RNA methylation, the most common modification of messenger RNAs (mRNAs), influences many steps of mRNA metabolism, including splicing, export, and translation, as well as stability [9]. Methyltransferase‐like 14 (METTL14), a core component of the multicomponent methyltransferase complex (MTC), colocalizes with Methyltransferase‐like 13 (METTL13) and Wilms tumor 1–associated protein (WTAP) in nuclear speckles and catalyzes the m^6^A modification [10]. In contrast, the fat mass and obesity–associated protein (FTO) and alkB homolog 5 (ALKBH5) act as m^6^A demethylases, catalyzing the removal of methyl groups from m^6^A‐modified RNAs [11]. Thus, m^6^A modification—governed by the functional crosstalk between methyltransferases and demethylases—is inherently dynamic and reversible [12]. Accumulating evidence has demonstrated that dysregulated m^6^A modification in mRNAs or noncoding RNAs exerts a pivotal function in tumorigenesis and malignant progression across diverse cancer types [13, 14, 15]. In recent years, RNA methylation has been shown to play an important role in regulating tumor angiogenesis [16, 17]. In renal cell carcinoma, METTL14‐mediated m^6^A modification promotes the stability of the TRAF1 mRNA in an IGF2BP2‐dependent manner, thereby significantly promoting tumor angiogenesis and sunitinib resistance [18]. In lung adenocarcinoma, IGF2BP2, an m^6^A reader protein, enhances FLT4 mRNA stability by recognizing m^6^A‐modified sites, subsequently activating the PI3K‐Akt signaling pathway and ultimately driving tumor angiogenesis and metastatic progression [17]. However, the functional roles of m^6^A modification in HCC angiogenesis remain obscure.

METTL14 regulates RNA stability and translation via m^6^A methylation [10], while Vascular endothelial growth factor‐A (VEGFA) is a key driver of HCC angiogenesis [19]. In tumors, VEGFA is produced by hypoxic tumor cells, endothelial cells (ECs) and infiltrating myeloid cells such as “M2” phenotype macrophages and neutrophils. Emerging evidence suggests m^6^A modification of VEGFA mRNA may modulate its expression [20], but the role of METTL14 in this context remains unexplored. Given METTL14's dysregulation in HCC and VEGFA's clinical significance, elucidating their interplay could reveal novel anti‐angiogenic strategies.

In this study, we explored the m^6^A modification of VEGFA and its regulatory mechanisms in HCC. We found that METTL14 is downregulated in HCC tissues, while VEGFA is upregulated, exhibiting a significant negative correlation that correlates with poor clinical prognosis. Functionally, METTL14 modulates VEGFA via m^6^A modification: overexpression of METTL14 suppresses HCC cell proliferation, invasion, and angiogenesis (evidenced by reduced tube formation in HUVECs and diminished vascularity in xenograft tumors), whereas METTL14 knockdown exerts opposite effects. These findings elucidate a novel METTL14‐m^6^A‐VEGFA axis governing HCC angiogenesis and progression, providing new mechanistic insights and potential therapeutic targets for anti‐angiogenic therapy in HCC.

## 2 Methods and Materials

### 2.1 Clinical Samples

HCC tissues and corresponding adjacent normal tissues were obtained from the sample bank of The Second Hospital of Nanjing. Upon removal from the body, tissue samples were immediately frozen in liquid nitrogen and stored at −80°C for further research. All tissue samples included in this study were pathologically confirmed by two pathologists. The research was performed in accordance with the guiding principles of the World Medical Association Declaration of Helsinki and was approved by the Ethics Committee of The Second Hospital of Nanjing (2023‐LS‐ky‐051). Clinical tissue samples were collected from [March, 2022] to [May, 2023]. In vitro cell experiments were conducted between [January, 2023] and [May, 2025]. Written informed consent was obtained from all patients (or their legal guardians) for the use of surgically resected tissues in this study, and patients were informed of the study's purpose, risks, and right to withdraw.

### 2.2 Cell Culture

Human HCC cell lines: SK‐Hep1 (SCSP‐526), Hep3B (HB‐8064), HepG2 (HB‐8065) were purchased from the American Type Culture Collection (ATCC, Rockefeller, MD, USA). Huh7 (SCSP‐526) was purchased from the Chinese Cell Bank of the Chinese Academy of Sciences (Shanghai, China), and MHCC97H (C6585), LM3 (C6303) were purchased from Beyotime Biotechnology (Shanghai, China). Liver normal epithelial cell line: THLE‐2 (CL‐0833) was purchased from Pricella Biotechnology (Wuhan, China). HUVEC (PCS‐100‐010) were also purchased from the American Type Culture Collection (ATCC, Rockefeller, MD, USA). All cells were incubated in an incubator with 5% CO_2_ at 37°C. The medium used for HCC cell lines was 89% Dulbecco's modified Eagle's medium (DMEM, 11965092, Gibco, USA) +10% fetal bovine serum (FBS, BI, Israel) +1% penicillin/streptomycin (15 140 122, Gibco, USA). The medium used for THLE‐2 was special medium (CM‐0833, Procell, Wuhan, China). The medium used for HUVEC was Endothelial cell medium (1001, ECM, Sciencell, USA).

All cell lines used in this study have been rigorously validated for authenticity via STR profiling and tested negative for mycoplasma contamination before use and during long‐term culture. The number of cells seeded in functional assays was listed in Table 1. All cell lines were used at low passage numbers (P3–P10) to maintain genetic stability, as recommended by ATCC guidelines.

**TABLE 1:** Sequences of primers.

Name	Primer sequences (5′– 3′)
*METTL14*	
Forward	AGTGCCGACAGCATTGGTG
Reverse	GGAGCAGAGGTATCATAGGAAGC
*VEGFA*	
Forward	AGGGCAGAATCATCACGAAGT
Reverse	AGGGTCTCGATTGGATGGCA
*GAPDH*	
Forward	GGAGCGAGATCCCTCCAAAAT
Reverse	GGCTGTTGTCATACTTCTCATGG

### 2.3 m^6^A RNA Methylation Quantification

Firstly, the total RNA in the tissues was extracted using the Trizol method (RC401‐01, Vazyme, China). Total mRNA was purified using the Dynabeads mRNA Purification Kit (61 006; Thermo Fisher Scientific). Then, DEPC (negative control), PC (0.5 ng/μL, 2 μL), and total mRNA (200 ng dissolved in 1–8 μL DEPC‐treated water) were immobilized onto the microplate surface following the manufacturer's instructions for the EpiQuik m^6^A RNA Methylation Assay Kit (P‐9005, Epigentek, USA). Specific m^6^A capture antibody, detection antibody, and color development reagent were added sequentially; the final color shade shown was proportional to the concentration of m^6^A in the samples. Finally, the absorbance (OD) was measured at 450 nm using an enzyme standardization instrument. The percentage of m^6^A in total RNA was calculated using the following formula.

$$\begin{array}{c} m^{6}A\%=\left[\left(\text{Sample}\;\mathrm{OD}-\mathrm{NC}\;\mathrm{OD}\right)÷200\;\mathrm{ng}\right]\\ \;÷\left[\left(\mathrm{PC}\;\mathrm{OD}-\mathrm{NC}\;\mathrm{OD}\right)÷1\;\mathrm{ng}\right]\times 100\% \end{array}$$

### 2.4 Cell Counting Kit‐8 (CCK‐8)

Cell viability was assessed using the Cell Counting Kit‐8 (CCK‐8) assay. Briefly, Huh7 cells were seeded in 96‐well plates at a density of 5 × 10^3^ cells/well and cultured in complete medium (DMEM supplemented with 10% fetal bovine serum, FBS) at 37°C in a humidified atmosphere containing 5% CO_2_. At 24, 48, 72, and 96 h post‐seeding, 10 μL of CCK‐8 reagent (Beyotime Biotechnology Co. Ltd., Shanghai, China) was added to each well. After incubation for 1–2 h at 37°C, the absorbance (optical density, OD) was measured at 450 nm using a microplate reader. Each experiment was performed in triplicate.

### 2.5 Colony Formation Assay

For the colony formation assay, Huh7 cells were seeded at a density of 5 × 10^2^ cells/well in six‐well plates and cultured for 14 days at 37°C in a humidified atmosphere containing 5% CO_2_. After incubation, the cultures were fixed with 4% paraformaldehyde, stained with 1% crystal violet, and washed. The number of colonies was then counted, and representative images were captured.

### 2.6 Transwell Assay

Cell invasion was assessed using Transwell chambers (Corning, NY, USA). Briefly, Huh7 cells were trypsinized, resuspended in serum‐free DMEM at a density of 5 × 10^4^ cells/mL, and seeded into the upper chambers of Transwell inserts pre‐coated with Matrigel (BD Biosciences, Franklin Lakes, NJ, USA). The lower chambers were filled with DMEM supplemented with 10% FBS as a chemoattractant. After incubation for 24 h at 37°C in a humidified atmosphere containing 5% CO_2_, non‐invaded cells on the upper surface of the membrane were gently removed with a cotton swab. Invaded cells adhering to the lower surface of the membrane were fixed with 4% paraformaldehyde, stained with 0.1% crystal violet for 15 min, and washed with PBS. The number of invaded cells was manually counted under an inverted microscope (Zeiss, Germany) in five random fields per membrane, and representative images were captured.

### 2.7 Angiogenesis Assay

Firstly, low concentration of matrix gel (082704, ABW, China) was added to a 24‐well culture plate, after the matrix gel solidified, HUVEC cells at logarithmic growth stage were taken, counted, and inoculated on matrix gel at approximately 1 × 10^5^ per well. Finally, the supernatant of HCC cells collected after 48 h of transfection was added, and the cells were cultured for about 6 h in total. The morphology of the cells into tubes was observed under the microscope and photographed.

### 2.8 Quantitative Real‐Time PCR

Total cellular and tissue RNA was extracted using FastPure Cell/Tissue Total RNA Isolation Kit V3 (RC122‐01, Vazyme, China). The complementary DNA (cDNA) was synthesized by HiScript II Q RT SuperMix for qPCR kit (R223‐01, Vazyme, China). The relative mRNA expression levels of genes were determined using the ChamQ SYBR qPCR Master Mix kit (Q341‐02, Vazyme, China). Then calculated using the 2^ΔΔ^Ct method. GeNorm algorithm (v2.0) was used to validate reference gene stability (M < 0.5 in all samples). The sequence of primers used is shown in Table 2.

**TABLE 2:** Clinical characteristics of HCC patients.

Characteristics	Expression of METLL14		*p*
	High No. cases (%)	Low No. cases (%)	Fisher's exact test
Age (years)			
< 50	5 (41.7)	4 (23.5)	0.4223
≥ 50	7 (58.3)	13 (76.5)	
Gender			
Male	10 (83.3)	14 (82.4)	> 0.9999
Female	2 (16.7)	3 (17.6)	
ES stage			
I + II	6 (46.2)	1 (6.25)	0.0260*
III + IV	7 (53.8)	15 (93.75)	
AFP			
< 13.6	6 (50.0)	10 (58.8)	
≥ 13.6	6 (50.0)	7 (41.2)	
BCLC stage			
A–B	7 (53.8)	4 (25.0)	0.1426
C–D	6 (46.2)	12 (75.0)	
Degree of inflammation			
G1–G2	11 (91.7)	10 (58.8)	0.0926
G2–G3	1 (8.3)	7 (41.2)	
Degree of fibrosis			
S1–2	3 (27.3)	4 (23.5)	> 0.9999
S3–4	8 (72.7)	13 (76.5)	

Abbreviations: AFP, alpha‐fetoprotein; BCLC, Barcelona Clinic Liver Cancer; ES, Edmondson‐Steiner.

* *p* < 0.05.

### 2.9 Western Blot

Protein samples were made by lysing cell precipitates using RIPA lysate, then mixed with 5× sample loading buffer, denatured by boiling in a 95°C water bath, and cooled on ice. The samples were separated by 12% SDS‐PAGE and transferred to PVDF membranes (0.45 μm, Millipore, USA). To ensure consistency, GAPDH and test proteins were detected on the same PVDF membrane. After electrophoresis, GAPDH (36 kDa) and METTL14 (55 kDa) are located in different regions of the same membrane. We cut the membrane into strips corresponding to these molecular weight ranges. Each strip was then separately blocked and incubated with the respective primary and secondary antibodies. GAPDH (36 kDa) and VEGFA (36 kDa) are located in the same regions of the membrane. GAPDH was detected on the same membrane after stripping and re‐probing. Unspecific proteins were blocked with non‐fat milk for 2 h. Then the membrane was incubated with primary antibody for 2 h, which was co‐incubated overnight at 4°C with primary antibody (GAPDH, CL594‐60004, Proteintech, China; anti‐METTL14, 26 158‐1‐AP, Proteintech, China; anti‐VEGFA, A12303, ABclonal, China), and followed by correspondingly secondary antibodies for 1 h at room temperature. Finally, the membrane was exposed using the ultrasensitive luminescence Kit (P0018FS, Beyotime, China).

Western blot bands were quantified using ImageJ software (v1.53t, NIH, USA). Relative protein levels were normalized to the internal control (GAPDH) and expressed as fold‐changes relative to the control group. Statistical significance was assessed using Student's *t*‐test. Data are presented as mean ± SD from three independent experiments (*n* = 3). A *p*‐value < 0.05 was considered statistically significant, with asterisks indicating: * *p* < 0.05, ** *p* < 0.01, ****p < 0.001.

### 2.10 Immunohistochemical Staining

Pathological sections for immunohistochemical sectioning were provided by The Second Hospital of Nanjing. HCC tissue and paraneoplastic tissue were fixed in 4% paraformaldehyde, dehydrated and embedded in an embedding box, and then the tissue was sectioned into 5 μm thickness. Dewaxed and hydrated at room temperature, samples were subjected to antigenic thermal repair in citric acid buffer, cooled and repeatedly washed with buffer, diluted serum was added to close the non‐specific sites (37°C incubator, 30 min). After serum removal, METTL14 (1:200, 80 790‐1‐RR, Proteintech, China), CD31 (1:200, 66 065‐2‐Ig, Proteintech, China) primary antibody was added and incubated overnight at 4°C. The next day, rabbit secondary antibody was added and incubated for 1 h at room temperature, finally stained with DAB Peroxidase Substrate Kit (MM1702‐1 KIT, MKBio, China) for observation. The staining results were evaluated by pathologists who were blinded to the clinical data. The staining intensity was scored as 0 (negative), 1 (weak), 2 (moderate), or 3 (strong). The percentage of positive cells was categorized as 0 (< 5%), 1 (6%–25%), 2 (26%–50%), 3 (51%–75%), or 4 (> 75%). The final IHC score was calculated as: Intensity Score × Percentage Score.

### 2.11 Animal Experiments

All the animals used in this study were kept in a pathogen‐free environment and fed properly. The procedures for care and use of animals were approved by the Ethics Committee of the Nanjing University of Chinese Medicine (22‐270) and all applicable institutional and governmental regulations concerning the ethical use of animals were followed. For the tumorigenesis assay, 5‐week‐old female BALB/c‐nu mice (16–20 g initial body weight) were selected and randomly divided into the pc‐DNA3.1 group and the METTL14 overexpress group. Huh7 cells were transfected with pc‐DNA3.1 or pCMV3‐GFP‐Puro‐METTL14 plasmids. The cells were collected at 48 h after transfection, and resuspended with a high concentration of matrix glue (0827245, ABW, China) and injected subcutaneously into the left or right back of mice. Each animal was injected with about 2 × 10^7^ cells/200 μL and sacrificed after 21 days. Body weight was measured every 3 days using a digital scale (Sartorius, Germany) and recorded until the endpoint. At sacrifice, final body weights were: pc‐DNA3.1 group (23.3 ± 1.2 g) and pCMV3‐ METTL14 group (25.6 ± 1.5 g). The tumor volume was measured by caliper twice weekly (length [L], width [W]). The tumor volume calculated as V = (L × W^2^)/2. Tumor morphology, and angiogenesis of subcutaneous tumors were observed.

### 2.12 MeRIP‐qPCR

The Magna MeRIP m^6^A Kit (A‐17‐10 499‐1, Merck, Germany) was used to monitor the status of m^6^A and map the location of m^6^A VEGFA RNA modifications transcriptome‐wide. Firstly, total RNA from HCC cell lines was collected. Fragmentation buffer was added and lysed at 94°C for 4 min, and EDTA was added immediately; this step fragmented the RNA. Two microliters of the RNA sample were designated as the input control. Twenty microliters of RNA sample were separately incubated with 4 μg of anti‐m^6^A antibody or isotype‐matched IgG control antibody overnight (16 h) at 4°C on a vertical rotator. Subsequently, 20 μL of protein A/G magnetic beads were added to each immunoprecipitation (IP) reaction—anti‐m^6^A and IgG—and incubated for 2 h at 4°C on the same vertical rotator. Following incubation, samples were placed on a magnetic rack for 1 min to separate beads from supernatant; the supernatant was carefully removed. Each bead pellet was then washed three times with 200 μL of ice‐cold washing buffer: after gentle vortexing to resuspend beads, samples were rotated for 5 min at 4°C, followed by magnetic separation for 1 min and supernatant removal after each wash. Finally, RNA was eluted from the input, m^6^A‐IP, and IgG‐IP samples, followed by reverse transcription. Enrichment of m^6^A modifications at distinct regions of the VEGFA transcript was quantified by quantitative real‐time PCR using region‐specific primers.

### 2.13 RNA Pull‐Down Assays

To validate the direct interaction between METTL14 and VEGFA mRNA. Biotin‐labeled RNA probes targeting VEGFA mRNA m^6^A‐enriched regions were synthesized. Total protein was extracted from Huh7 cells, incubated with probes, and protein‐RNA complexes were captured using streptavidin magnetic beads. Bound proteins were detected by Western blot (anti‐METTL14 antibody), with GAPDH as loading control.

### 2.14 Statistical Analysis

For data on HCC‐related gene expression, correlation, and patient survival, statistics were obtained from a portal site based on TCGA, GEO database (http://kmplot.com/), one‐way ANOVA, and two‐sided independent *t*‐test were applied to process the data, ImageJ software was used to count the image data, and image drawing and analysis with GraphPad Prism 12.0 software. All experiments were independently repeated three times or more.

## 3 Results

### 3.1 Low METTL14 Expression in HCC Correlates With Patient Prognosis

**FIGURE 1:**
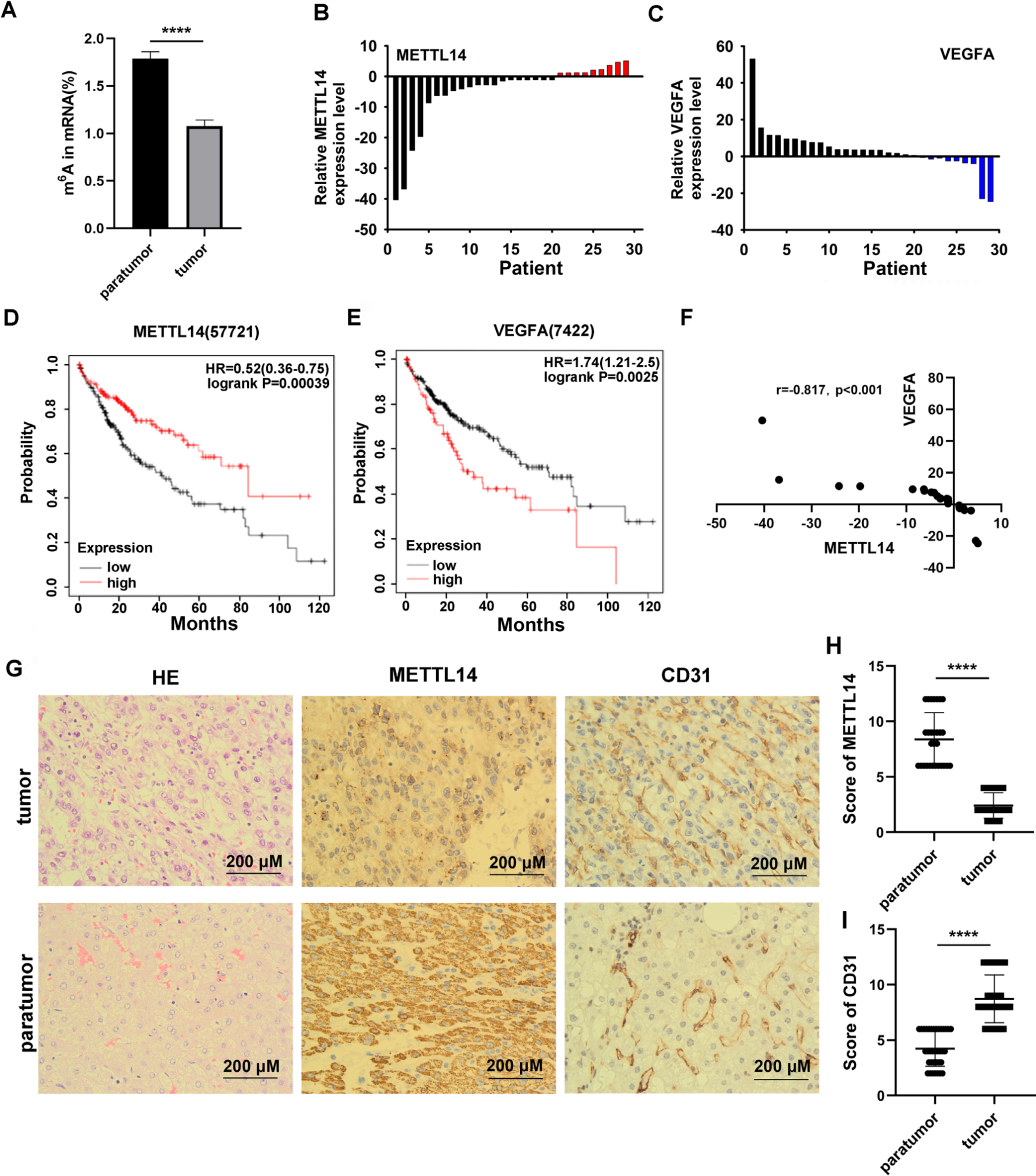
Expression of METTL14 and VEGFA in HCC and their clinical significance. (A) m^6^A levels in mRNA from paired hepatocellular carcinoma (HCC) and paracancerous tissues (*n* = 10 pairs; mean ± SD; ****p < 0.0001 vs. paracancerous tissues, paired *t*‐test). (B) METTL14 mRNA and (C) VEGFA mRNA relative expression in 29 HCC patients. When the expression level of the tumor tissue is higher than that of the adjacent tissue, divide the relative mRNA expression level of the tumor tissue by that of the adjacent tissue and represent it with a positive number. When the expression level of the tumor tissue is lower than that of the adjacent normal tissue, divide the relative mRNA expression level of the adjacent normal tissue by that of the tumor tissue and represent it with a negative number. (D) Kaplan–Meier overall survival (OS) analysis of 364 HCC patients stratified by METTL14 expression (low: *n* = 204, high: *n* = 160; median split; log‐rank test, *p* < 0.001). (E) Kaplan–Meier OS analysis of 364 HCC patients stratified by VEGFA expression (low: *n* = 268, high: *n* = 96; median split; log‐rank test, *p* < 0.01). (F) Correlation between VEGFA and METTL14 mRNA expression in 29 paired HCC tissues (Pearson *r* = −0.817, *p* < 0.001). (G) Representative immunohistochemical (IHC) staining of METTL14 and CD31 in matched HCC and paracancerous tissues (scale bar: 200 μm). Statistical results for METTL14 (H) and CD31 (I) expression in matched HCC and paracancerous tissues. (*n* = 29 pairs; mean ± SD; ****p < 0.0001, paired *t*‐test).

The expression of the overall level of m^6^A was first examined in the tissues, and the results showed that the level of m^6^A in HCC tissues was lower than that of the paracancer (Figure 1A). qPCR experiments on 29 pairs of HCC surgical samples and paired paracancer samples, revealed that the mRNA level of METTL14 was significantly downregulated in the cancer tissues (Figure 1B). While VEGFA was highly expressed in HCC tissues (Figure 1C).

In addition, using the online Kaplan–Meier Plotter database (Liver RNA‐seq version, kmplot.com), we analyzed RNA‐seq data from tumor tissues of 364 HCC patients, with 160 cases exhibiting high METTL14 expression and 204 cases showing low expression. At a time cutoff of 120 months (10 years), patients with high METTL14 expression demonstrated significantly longer overall survival compared to those with low expression (Figure 1D). These results indicate a positive correlation between METTL14 expression and patient prognosis in HCC: lower METTL14 expression is associated with poorer prognosis. Similarly, analysis of VEGFA mRNA expression levels in tumor tissues from these 364 HCC patients revealed 96 cases with high expression and 268 cases with low expression. At a time cutoff of 120 months (10 years), patients with high VEGFA mRNA expression demonstrated significantly shorter overall survival compared to those with low expression (Figure 1E). These results indicate a negative correlation between VEGFA mRNA expression and patient prognosis in HCC: higher VEGFA mRNA expression is associated with poorer prognosis. This finding is opposite to the positive correlation observed between METTL14 and prognosis in HCC in our previous analysis.

Moreover, correlation analysis showed a negative correlation between the expression of METTL14 and VEGFA in cancer tissues (Figure 1F). Analysis of patients' clinical data combined with experimental validation revealed that abnormally reduced METTL14 mRNA levels were significantly associated with low tumor differentiation, and this association was statistically robust. In contrast, no significant correlations were observed for gender, age, or liver fibrosis (Table 2). To validate these findings, paired hepatocellular carcinoma (HCC) and adjacent non‐tumor tissue sections from our clinical biobank underwent immunohistochemical (IHC) staining. The results revealed that METTL14 protein expression was markedly lower in HCC tissues compared to adjacent non‐tumor counterparts, whereas VEGFA exhibited an inverse expression pattern (Figure 1G–I). Collectively, these data indicate that low METTL14 expression (accompanied by reduced m^6^A levels) in HCC is typically associated with elevated VEGFA expression—a relationship that may critically contribute to malignant progression and poor prognosis in HCC.

### 3.2 METTL14 Overexpression Inhibited the Proliferation, Invasion, and Angiogenesis of HCC Cells In Vitro

**FIGURE 2:**
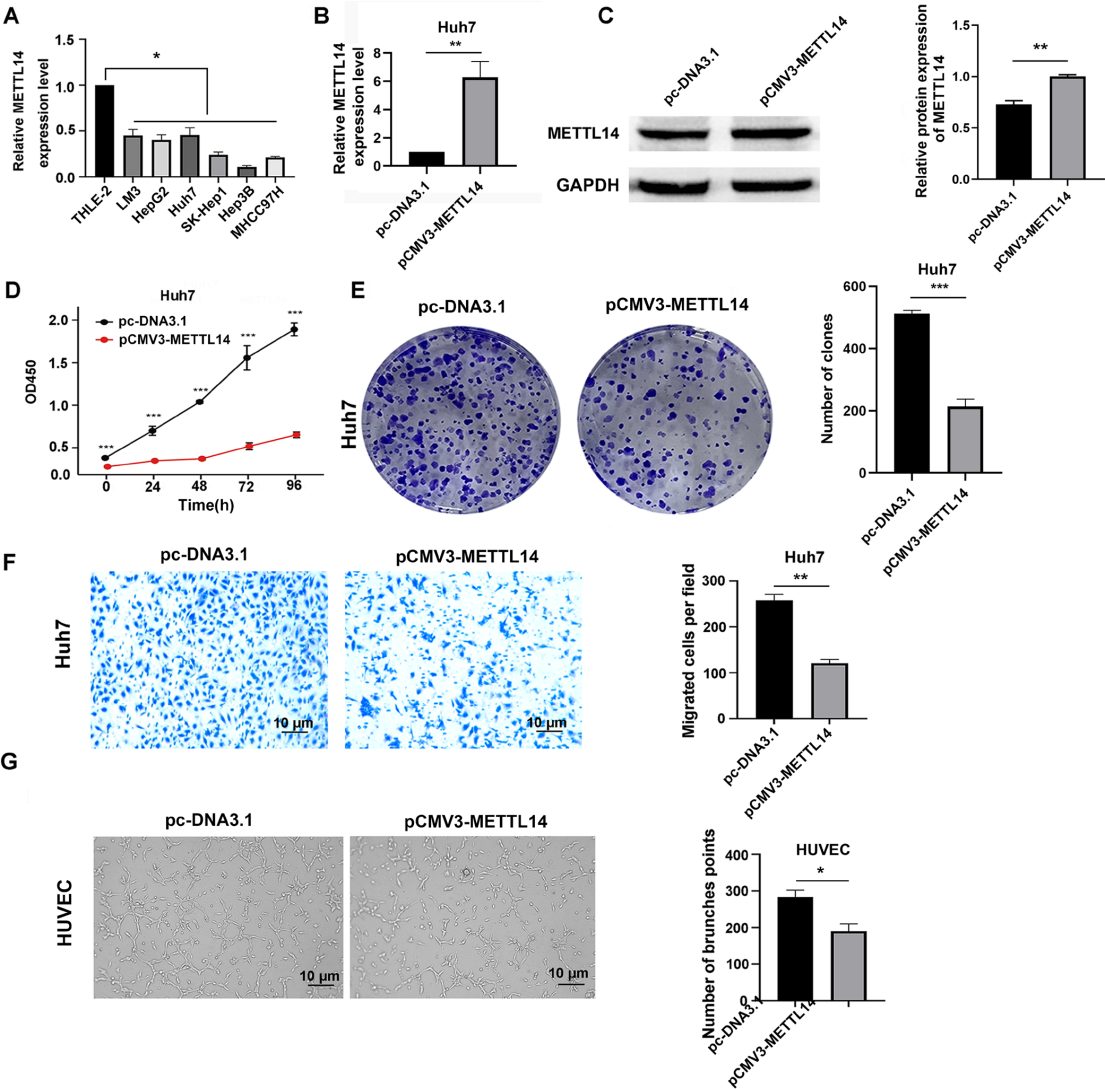
METTL14 overexpression inhibits proliferation, invasion of Huh7, and tube‐forming ability of HUVEC cells in vitro. (A) METTL14 mRNA expression in HCC cells (LM3, HepG2, Huh7, SK‐Hep1, Hep3B, MHCC97H) and normal liver cells (THLE‐2) (Data normalized to ACTB/HPRT1/GAPDH (geNorm‐validated); mean ± SD, *n* = 3; *p < 0.05 vs. THLE‐2, one‐way ANOVA). (B) qRT‐PCR analysis of METTL14 mRNA levels in Huh7 cells 48 h after transfection with METTL14 overexpression plasmid (pCMV3‐METTL14) or empty vector (pc‐DNA3.1) (mean ± SD, *n* = 3 independent experiments; ***p < 0.001 vs. Control, *t*‐test). (C) Western blot validation of METTL14 overexpression efficiency in Huh7 cells with GAPDH as loading control. (mean ± SD, *n* = 3 independent experiments; ***p < 0.001 vs. Control, *t*‐test). (D) CCK‐8 proliferation assay of Huh7 cells (pCMV3‐METTL14 vs. Control) over 24, 48, 72, and 96 h (mean ± SD, *n* = 3; ***p < 0.001 vs. Control, *t*‐test). (E) Colony formation assay of Huh7 cells (pCMV3‐METTL14 vs. Control) (mean ± SD, *n* = 3; ***p < 0.001 vs. Control, *t*‐test). (F) METTL14 overexpression inhibits Huh7 invasion via Matrigel‐coated Transwell assay. (Left) Representative light microscopy images of invaded cells (scale bar: 10 μm). (Right) Quantitative analysis of invaded cell numbers (mean ± SD, *n* = 3; **p < 0.01 vs. Control, *t*‐test). (G) Tube formation assay of HUVEC cells co‐cultured with conditioned medium from Huh7 cells (pCMV3‐METTL14 vs. Control) (scale bar: 10 μm). (Data: Mean ± SD, *n* = 3; *p < 0.05 vs. Control, *t*‐test).

To investigate the role of METTL14 in the malignant progression of HCC, HCC cell lines (LM3, HepG2, Huh7, SK‐Hep‐1, Hep3B, MHCC97H) were screened for METTL14 mRNA level using qPCR method. THLE‐2 is an immortalized normal human liver epithelial cell line transformed with SV40 large T antigen. METTL14 expression was downregulated in HCC cell lines relative to THLE‐2 (Figure 2A), a pattern consistent with the METTL14 downregulation observed in primary HCC tissues compared to adjacent non‐tumor tissues in our clinical cohort (Figure 1B). LM3, HepG2, and Huh7 with moderate METTL14 expression were selected for candidate subsequent overexpression and knockdown experiments to ensure measurable dynamic range of functional effects. LM3, a highly metastatic subline generated via three rounds of lung metastasis screening in nude mice, was derived from MHCC97‐H cells. HepG2, an embryonic liver‐derived hepatoblastoma line, differs from most HCC models. Huh7, retaining key epithelial traits of primary HCC and METTL14 expression aligned with clinical samples, was selected for functional experiments. Huh7 cells were transfected with pc‐DNA3.1 or METTL14 overexpression plasmid. qPCR assay was performed to detect the transfection efficiency (Figure 2B), and Western blot analysis showed an increase in METTL14 levels after transfection (Figure 2C). Then the proliferation ability of cells after overexpression of METTL14 was detected using CCK8 assay. The absorbance of cells at OD450 wavelength was measured at 0, 24, 48, 72, and 96 h using an enzyme standard, and it was found that the proliferation ability of cells in the experimental group was significantly inhibited compared to the control group (Figure 2D). The cell cloning assay also proved this result (Figure 2E). Matrigel‐coated Transwell assay results showed that overexpression of METTL14 significantly inhibited the invasion ability of HCC cells (Figure 2F). The culture medium supernatant of Huh7 cells was collected and used for HUVEC cells culture. It was found that compared with the pc‐DNA3.1 group, the number of HUVEC tube formation in the METTL14 overexpression group significantly decreased (Figure 2G). These results confirmed that overexpression of METTL14 could inhibit the growth and invasion ability of HCC cells in vitro, and reduce the formation of blood vessels in vitro.

### 3.3 METTL14 Knockdown Promotes HCC Cell Proliferation and Metastasis In Vitro

**FIGURE 3:**
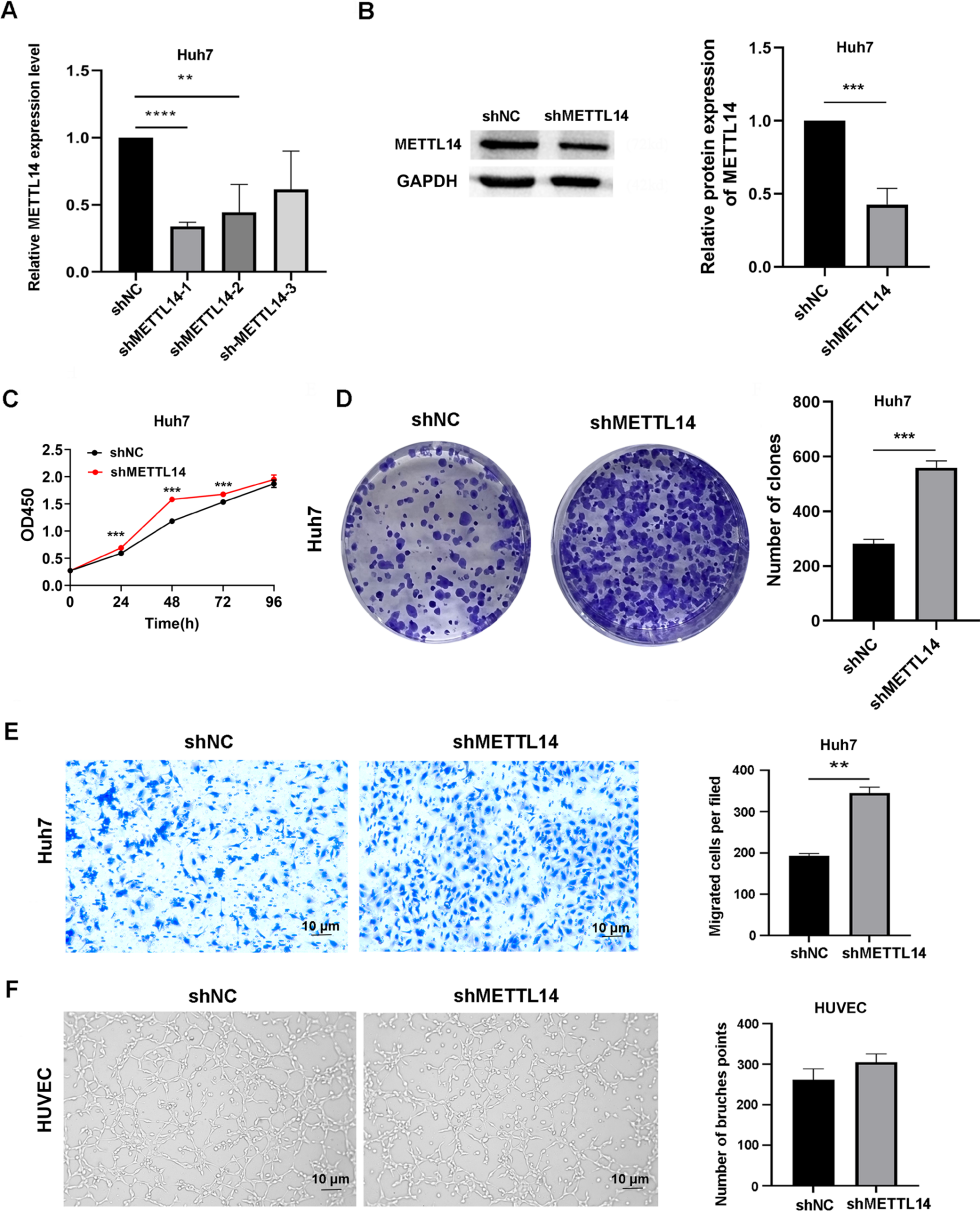
Knockdown of METTL14 promotes Huh7 cell proliferation and invasion. (A) shRNA‐mediated METTL14 knockdown in Huh7 cells. Short hairpin RNA (shRNA) targeting METTL14 (sh‐METTL14) or non‐targeting control shRNA (sh‐NC) was transfected into Huh7 cells to silence METTL14 expression. (B) Western blot validation of METTL14 knockdown efficiency in Huh7 cells with GAPDH as loading control (mean ± SD, *n* = 3; *p < 0.05 vs. sh‐NC, *t*‐test). (C) CCK‐8 proliferation assay of Huh7 cells (sh‐METTL14 vs. sh‐NC) over 24, 48, 72, and 96 h (mean ± SD, *n* = 3; ***p < 0.001 vs. sh‐NC, *t*‐test). (D) Colony formation assay of Huh7 cells (sh‐METTL14 vs. sh‐NC) (mean ± SD, *n* = 3; ***p < 0.001 vs. sh‐NC, *t*‐test). (E) Matrigel‐coated Transwell invasion assay of Huh7 cells (sh‐METTL14 vs. sh‐NC). Representative light microscopy images of invaded cells (scale bar: 10 μm). Quantitative analysis: Invaded cell number (mean ± SD, *n* = 3; **p < 0.01 vs. sh‐NC, *t*‐test). (F) Tube formation assay of HUVEC cells co‐cultured with conditioned medium from Huh7 cells (scale bar: 10 μm) (mean ± SD, *n* = 3; sh‐METTL14 vs. sh‐NC, ns, *t*‐test).

Firstly, Huh7 cells were transfected with shRNAs targeting METTL14, qPCR assay was performed to detect the transfection efficiency (Figure 3A), and Western blot analysis showed that the level of METTL14 was reduced after transfection (Figure 3B). CCK8 assay was used to access cell viability, and the results showed that METTL14 depletion could promote cell proliferation (Figure 3C), and the same results were observed in the cell colony formation assay (Figure 3D). Subsequent Matrigel‐coated Transwell assay showed that the invasion ability of Huh7 cells was improved when METTL14 was silenced (Figure 3E). In the angiogenesis assay, although the medium supernatant of METTL14 knockdown cells was used to culture HUVEC, the results were not as expected; the tube‐forming ability of HUVEC in the shMETTL14 group was not significantly different from that of the shNC group (Figure 3F). The above experimental results confirmed that the silencing of METTL14 could negatively regulate the malignant proliferation of HCC cells and promote invasion, but the effect on angiogenesis was not significant.

### 3.4 Overexpression of METTL14 Reduces Angiogenesis In Vivo

**FIGURE 4:**
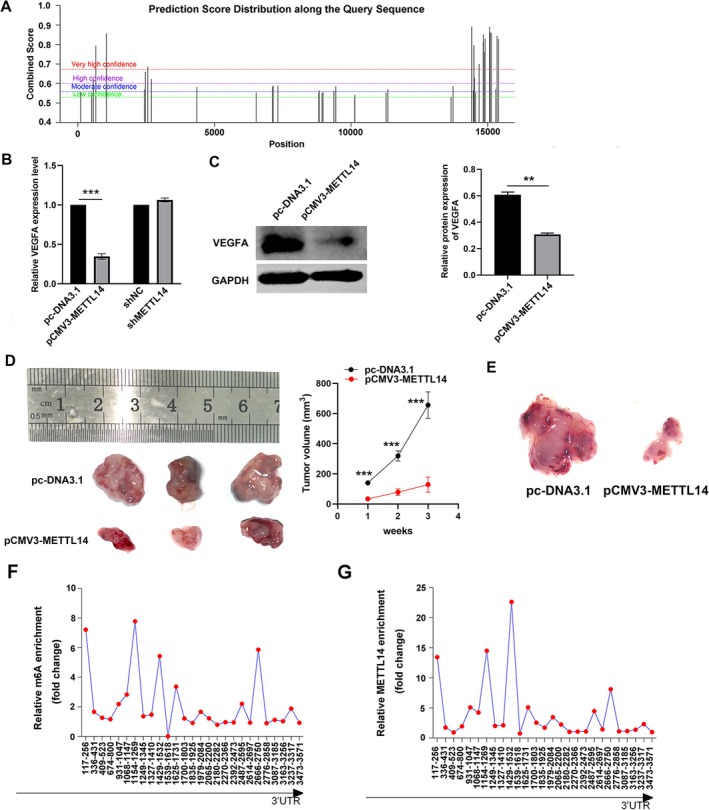
METTL14 regulates VEGFA expression via m^6^A modification and inhibits angiogenesis in vivo. (A) In silico prediction of multiple m^6^A modification sites across the full‐length VEGFA mRNA using SRAMP/RMBase databases (http://www.cuilab.cn/sramp). (B) qRT‐PCR analysis of VEGFA mRNA levels in HCC cells (Huh7) after METTL14 overexpression (pCMV3‐METTL14 vs. Control) or knockdown (sh‐METTL14 vs. sh‐NC) (Data normalized to ACTB/HPRT1/GAPDH (geNorm‐validated); mean ± SD, *n* = 3; *p < 0.05, ***p < 0.001 vs. Control/sh‐NC, *t*‐test). (C) Western blot detection of VEGFA protein expression in pCMV3‐METTL14 Huh7 cells (GAPDH as loading control; *n* = 3). (D) Comparison of subcutaneous tumor volume in nude mice (BALB/c‐nu, 5‐week‐old, *n* = 3/group) inoculated with pCMV3‐METTL14 or Control Huh7 cells (mean ± SD, *n* = 3; ***p < 0.001 vs. Control, *t*‐test). (E) Stereomicroscopic images of vascularization in subcutaneous tumors. (F) MeRIP‐qPCR validation of m^6^A enrichment on VEGFA mRNA in anti‐m^6^A vs. IgG. (G) RNA pull‐down assay confirming METTL14 enrichment on VEGFA mRNA (biotin‐labeled VEGFA probe vs. irrelevant probes; Western blot detection of METTL14).

The liver is a vascular‐rich visceral organ, and tumor malignant proliferation and recurrence are often associated with blood vessels. To determine whether the expression level of VEGFA is associated with m^6^A modification of its mRNA, we initially performed bioinformatics analysis using the online m^6^A site predictor SRAMP (http://www.cuilab.cn/sramp), which revealed that VEGFA mRNA harbors multiple m^6^A modification sites (Figure 4A). Subsequently, we examined VEGFA expression changes under varying METTL14 expression levels. The results demonstrated that overexpression of METTL14 led to a significant decrease in both VEGFA mRNA and protein levels, with the mRNA reduction being particularly pronounced. In contrast, upon METTL14 silencing, VEGFA mRNA expression remained largely unchanged, and no corresponding protein alteration was observed (Figure 4B,C). Based on the results of the in vitro angiogenesis assay, we next designed a subcutaneous tumorigenesis assay in nude mice to further validate this in vitro observation in vivo. Following inoculation of nude mice with METTL14‐overexpressing HCC cells (experimental group) or control cells (control group), tumor growth was monitored for 4 weeks until sacrifice at a consistent endpoint. Macroscopically, tumors from the experimental group were significantly smaller than those in the control group. Histopathological analysis further revealed reduced vascular infiltration in the experimental group tumors compared to controls, indicating impaired angiogenesis (Figure 4D,E). Subsequently, we performed MeRIP (m^6^A‐specific methylated RNA immunoprecipitation) and RNA pull‐down assays to characterize m^6^A modification and protein‐RNA interactions on VEGFA mRNA. These experiments revealed abundant m^6^A enrichment sites on VEGFA mRNA. Notably, METTL14 was significantly enriched on VEGFA mRNA, with its enrichment pattern aligning with the m^6^A distribution trend, and direct interaction sites between METTL14 and VEGFA mRNA were identified (Figure 4F,G).

**FIGURE 5:**
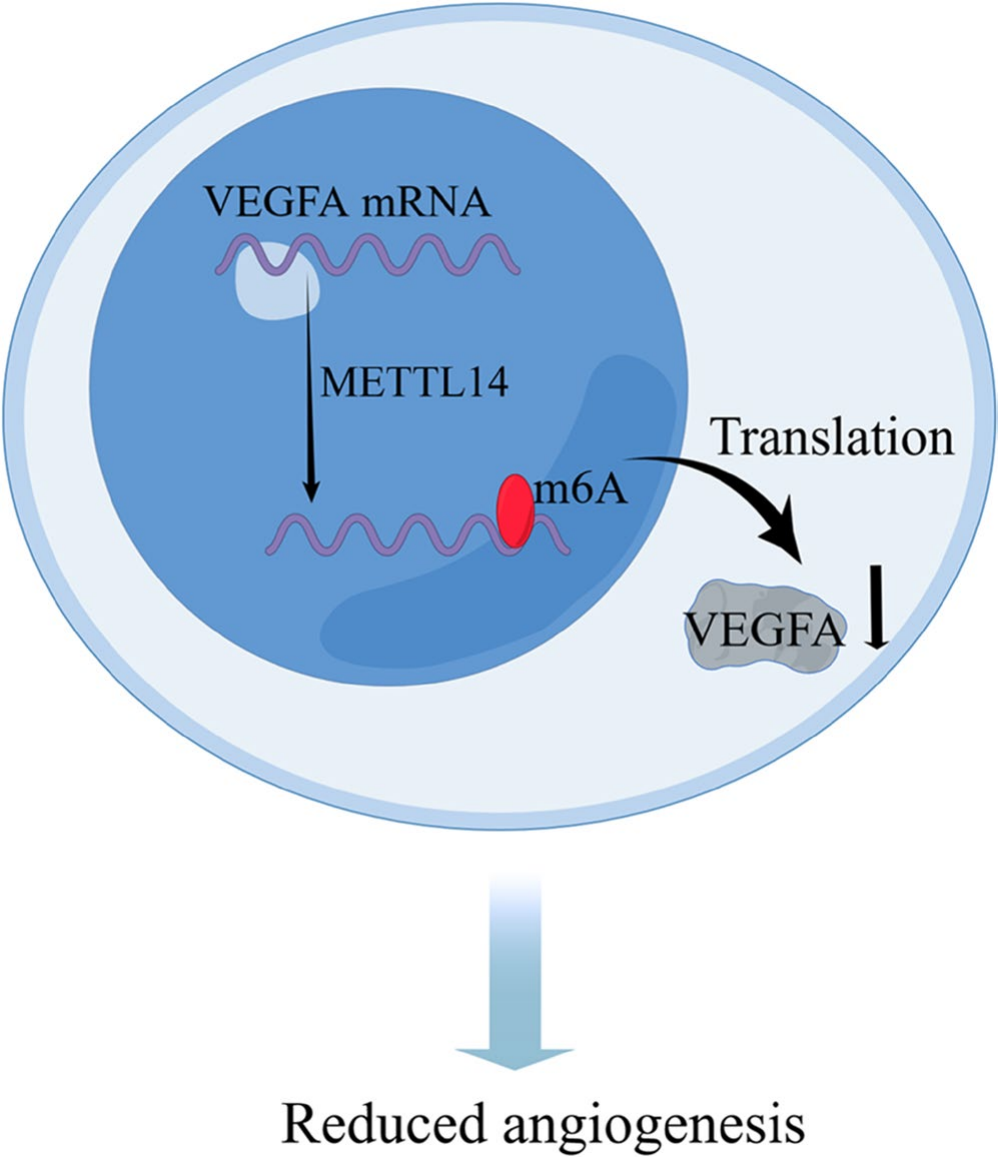
Schematic illustration of the mechanism by which METTL14 suppresses angiogenesis in hepatocytes via m^6^A‐mediated modification of VEGFA mRNA. In hepatocytes, METTL14 suppresses VEGFA expression via m^6^A methylation of VEGFA mRNA, thereby inhibiting angiogenesis. However, the downregulation of METTL14 in HCC leads to the upregulation of VEGFA, thereby promoting tumor angiogenesis.

Overall, METTL14 overexpression negatively regulated HCC angiogenesis in vivo and in vitro, resulting in less malignant tumor progression and metastasis, and METTL14 silencing promoted HCC proliferation and invasion, but had no significant effect on angiogenesis. Mechanistically, we propose that METTL14‐mediated m^6^A modification destabilizes VEGFA mRNA, reducing its secretion and suppressing angiogenesis in normal hepatocytes (Figure 5). However, the downregulation of METTL14 in HCC leads to the upregulation of VEGFA, thereby promoting tumor angiogenesis.

## 4 Discussion

In the study, m^6^A levels were low in HCC tissues, consistent with the expression of METTL14. According to data in the database, lower expression of METTL14 was correlated with shorter patient survival. On the contrary, higher METTL14 expression was correlated with longer overall survival in patients. qPCR results of our clinical HCC tissue samples revealed that the levels of METTL14 were lower in HCC tissues than that of para cancerous tissues, and the results of immunohistochemistry confirmed this conclusion. Moreover, METTL14 can negatively regulate the proliferation and invasion of HCC cells in vivo and in vitro.

m^6^A is the most prevalent internal modification of eukaryotic mRNA and plays a unique role in post‐transcriptional regulation of RNA. By functioning through a dynamic system comprising methyltransferases (writers, e.g., METTL3/METTL14), demethylases (erasers, e.g., FTO/ALKBH5), and reader proteins (e.g., YTHDFs, IGF2BPs), m^6^A regulates key RNA metabolic processes including splicing, stability, translation, and localization [21, 22, 23]. Dysregulation of this modification contributes to tumorigenesis by aberrantly modulating the expression of cancer‐related genes—either activating oncogenes or silencing tumor suppressors—thereby disrupting cellular homeostasis [24, 25, 26]. METTL14, a key component of methyltransferases, functions primarily as part of the METTL3/METTL14 binding subunit, anchoring multiple methyl groups on downstream targets, modifying the target molecule, and exerting the corresponding biological effects [27]. Furthermore, METTL14 plays different roles in different cancers, either as a pro‐ or oncogene, and can promote or suppress the development of hematopoietic malignancies and solid tumors [28]. For example, METTL14‐mediated m^6^A modification inhibits hematopoietic stem/progenitor cell differentiation and promotes leukemogenesis [29]. Rhapontigenin inhibits epithelial mesenchymal transition of bladder cancer cells through the FOXO3A/METTL14/VIMENTIN pathway [30]. Additionally, targeting histone deacetylase suppresses tumor growth through eliciting METTL14‐modified m6 A RNA methylation in ocular melanoma [31]. Together, these examples illustrate the context‐dependent complexity of METTL14's functions and mechanisms in tumorigenesis. We found that overexpression of METTL14 inhibited the malignant behavior of HCC cells both in vivo and in vitro, reducing their proliferative and invasive capacities. In contrast, knockdown of METTL14 enhanced these malignant phenotypes, a conclusion consistent with that reported by Shi et al. [32]. More significantly, the tube formation capacity of endothelial cells was negatively related to the expressions of METTL14. The experimental studies on the effect of METTL14 on angiogenesis in HCC demonstrate that RNA methylation may play an essential role in tumor anti‐vascular therapy. Overexpression of METTL14 resulted in a decrease in both mRNA and protein levels of VEGFA. Moreover, a remarkable decrease in the vascular neovascularization capacity of HUVEC cells. Due to the low expression of METTL14 in HCC cell lines, no significant difference for the cell function was observed after METTL14 knockdown. In the existing reports, there have been several studies on RNA methylation and organismal anti‐inflammation, tumor therapy, and chemotherapy resistance [33, 34, 35], but not many studies on anti‐angiogenic related therapy, our experiment fills this gap and demonstrates that METTL14 can negatively regulate angiogenesis in HCC. Nevertheless, in the study, we verified the phenomenal association between METTL14 and VEGFA but did not explore how METTL14 regulate VEGFA through m^6^A modification, therefore, the specific mechanism of their interaction to inhibit HCC needs further study.

The vasculature plays an indispensable role in hepatocellular carcinoma (HCC) progression and tumor recurrence. First‐line targeted therapies for HCC, including the multikinase inhibitors sorafenib and lenvatinib, primarily target vascular endothelial growth factor (VEGF) signaling to suppress angiogenesis at the tumor site, thereby exerting therapeutic efficacy [36]. In addition, the side effects of targeted drugs make many patients have to reduce the dosage, and drug resistance is also very common in clinical practice. HCC angiogenesis is closely related to the complex tumor microenvironment, with multiple mechanisms such as tissue hypoxia and immune microenvironment all involved in the regulation of angiogenesis [37, 38]. Faced with this dilemma, exploring novel anti‐angiogenic factors has emerged as an urgent and clinically relevant priority in our clinical cohort: VEGFA mRNA expression was significantly higher in HCC tissues compared to adjacent non‐tumor tissues. Accumulating evidence indicates that elevated VEGFA expression contributes to tumor angiogenesis. Furthermore, a machine learning model constructed based on genes highly expressed in VEGFA‐positive cancer‐associated fibroblasts (CAFs) exhibited robust predictive accuracy for forecasting prognosis and sorafenib therapeutic response among patients with HCC [39].

Currently, our study has only revealed a correlative link between METTL14, m^6^A modification, and VEGFA mRNA levels. Identifying the specific m^6^A reader protein(s) that bind to VEGFA mRNA is critical for elucidating the mechanistic basis of this regulation, as it would directly connect m^6^A deposition by METTL14 to downstream functional outcomes (e.g., mRNA stability or translation). However, given the primary focus of this work—establishing METTL14 as a tumor‐suppressive regulator of HCC angiogenesis via the m^6^A‐VEGFA axis—a detailed dissection of reader proteins was beyond the scope of the current study. We plan to address this in follow‐up research, where we will employ RNA immunoprecipitation (RIP) assays with candidate readers (e.g., YTHDF family, IGF2BPs) and functional validation (e.g., reader knockdown/rescue experiments) to definitively identify the VEGFA m^6^A reader and characterize its role in HCC pathogenesis.

While our data implicate the METTL14‐VEGFA axis in HCC angiogenesis, we acknowledge potential confounders. For example, METTL14 loss may indirectly alter hypoxia signaling (e.g., HIF‐1α stabilization) or inflammatory cytokines (e.g., IL‐6), which could synergize with VEGFA inhibition. Additionally, partial METTL14 knockdown might insufficiently disrupt m^6^A methyltransferase activity in certain contexts. Future studies using CRISPR‐Cas9‐mediated knockout or tissue‐specific Mettl14 deletion in genetically engineered mouse models (GEMMs) will clarify these nuances.

In conclusion, METTL14 negatively regulates HCC proliferation and invasion. Its overexpression suppresses HCC angiogenesis by targeting VEGFA via m^6^A modification, thereby inhibiting tumor growth, reducing mortality and recurrence rates, and prolonging survival. Collectively, our findings position METTL14 as a promising therapeutic target for HCC angiogenesis, supporting further investigation into m^6^A‐based combination therapies.

## Author Contributions

**Jiayan Li:** investigation, resources, funding acquisition. **Lili Wang:** supervision, funding acquisition, writing – review and editing. **Xinmiao Xiong:** conceptualization, methodology, validation, formal analysis, writing – original draft. **Ziqun Qu:** methodology, validation, formal analysis. **Wei Ye:** conceptualization, funding acquisition, writing – review and editing. **Chuanfang Shu:** software, validation, formal analysis.

## Funding

This work was supported by the Jiangsu Commission of Health (grant number: H2023092, to L.L.W.); the Medical Science and Technology Development Foundation, Nanjing Municipality Health Bureau (grant number: YKK24179, to L.L.W.); the Jiangsu Provincial Cadre Health Care Research Project (grant number: BJ25045, to L.L.W.); The Second Hospital of Nanjing, Talent Promotion project (grant number: RCZD230004, to L.L.W.); the Foundation of The Clinical Infectious Disease Center of Nanjing (grant number: CXZX202232, to W.Y.); the Medical Science and Technology Development Foundation, Nanjing Municipality Health Bureau (grant number: YKK22132, to J.Y.L.); The Second Hospital of Nanjing, Reserve talents project (grant number: HBRCYL06, to J.Y.L.).

## Ethics Statement

This study was performed in accordance with the ethical standards as laid down in the 1964 Declaration of Helsinki and its later amendments or comparable ethical standards. The study was approved by the ethics committee of the second hospital of Nanjing (2023‐LS‐ky‐051).

## Consent

The patients were informed in writing.

## Conflicts of Interest

The authors declare no conflicts of interest.

## Data Availability

The data that support the findings of this study are available from the corresponding author upon reasonable request.
